# Monthly Summary

**Published:** 1876-02

**Authors:** 


### MONTHLY SUMMARY.

---

*Treatment of Neuralgia.*—The *Practitioner* gives the following from Dr. Von Pitha on this subject :—

V. Pitha himself has suffered for two years from the most severe neuralgia, in consequence of an accidental wound in an operation, and his account of his own case contains many points of interest. In particular he dwells upon the vagaries of the imagination in this affection, and states that he was perfectly convinced he had a stone in the bladder, the irritation of the projecting spiculœ as well as the blows of the stone against the mucous lining of the viscus being distinctly felt; and he was only satisfied of the incorrectness of his opinion after the bladder had been carefully searched with a sound. From that moment all the sensations vanished. From observations on himself and others, he believes the subcutaneous injection of morphia to be the most satisfactory mode of administering it, since it operates quickly, and the narcosis is slight compared with the great diminution of pain that is effected; moreover, it may be frequently repeated without injurious effects. The smallest effective doses should be commenced with, and these should only be increased when absolutely requisite. He gives a caution against employing morphia in those who have disease of the heart, as it sometimes produces in them deadly pallor, cold sweats, etc. When the toxic influence of morphia is manifested he recommends energetic walking in the open air, washing of the skin with vinegar, clysters of strong coffee, and, in extreme cases, artificial respiration. As a remedy preventing the unpleasant after-effects of morphia on the stomach and intestinal canal, V. Pitha recommends hydrochlorate of quinine, or a minute proportion (100th of a grain) of atropia may be added to the injection fluid.

*Subcutaneous Injection of Chloroform in the Treatment of Facial Neuralgia.*—Geo. Wood, M. D., C. M., writes as follows:

Dr. C. H., aged about fifty, dark hair, eyes and complexion, very spare, weight one hundred and twenty-five pounds, has suffered from facial neuralgia of left side of the face since 1854; has undergone all systems of treatment with little or no relief. In 1872 had the lower jaw trephined and a section of the nerve removed; this gave him relief for several months, but eventually the neuralgia returned harder, if possible than ever. He first came under my care in August, 1873. I gave him different irons, tonics, bark, hypodermic injections of morphia, croton chloral hydrate, and also all the different and various neuralgic pills that I ever heard of, with but temporary success.

In August of 1874, I injected fifteen minims of chloroform underneath the mucous membrane of the lower jaw, as near the exit of the mental branch of the fifth pair as I could. It gave him entire relief in an hour, but caused partial paralysis of the muscles of the left cheek. In a week I repeated the injection and put him upon drachm doses of the elix. of guarana three times a day. In September and December, and also in April, he had a very slight return of the pain, each recurrence being less severe. At each of these times I repeated the chloroform, and he now seems to be entirely well.

My partner, Dr. Rose, had a lady patient, unmarried, aged about forty-five, who had been a terrible sufferer from the same disease for seventeen years; had consulted the most eminent men in this and foreign countries, and had tried all the prescriptions recommended. The only thing that gave her any relief was the hypodermic injection of morphia. She was obliged to use them, sometimes several times a day. Her mind and morals were very much disordered. Dr. Rose tried the hypodermic use of chloroform, and she is now entirely well. Her mind is as clear as of yore, and she seems and acts like a new being. The injection of chloroform is extremely painful unless preceded by an injection of ten or fifteen minims of Magendie's sol. of morphia. If this suggestion can give relief to others suffering from this most painful disease I shall be most happy. I intend using the chloroform in the first case of sciatica that comes into my hands.—*Canada Med. and Surg. Journal.*

---

*Antidote to Carbolic Acid.*—A German contemporary affirms that the best antidote to carbolic acid is saccharate of lime, obtained by the solution of sixteen parts of sugar in forty parts of water, to which is added five parts of caustic lime. The solution should be filtered and evaporated to dryness. It is then easily soluble—*The American Medical Weekly.*

*Plastic Operation for the Lower Lip.*—In the *Transactions* of West Virginia, Dr. M. F. Hullihem, of Wheeling, gives an account of a plastic operation for the restoration of the lower lip, in the person of a soldier, who had lost the lip by gunshot wound. Measuring vertically, about one half of the lower maxillary bone, from the second bicuspid tooth on the left side to the first molar tooth on the right side, together with all the intervening teeth, had been shot away.

I made incisions, one inch and a quarter in length, carried in a straight line from each commissure of the mouth, directly into and entirely through the substance of each cheek. From the outer extremity of these incisions, another incision was made obliquely downward to the margin of the orifice, thus forming a triangular flap in each angle of the mouth. The mucous membrane lining the flaps thus formed, including a proper amount of structures beneath to insure vitality, were, save at the point of their lower attachment, carefully dissected away, after which the flaps were removed. The bases of these flaps pointed directly to each angle of the mouth, and measured in width (with a proper allowance for shrinkage) the distance it was necessary to raise the portion of chin out of which it was proposed to form a new lip.

After the bleeding ceased, an incision was made on the inside margin of the callous tissue forming the base of the orifice, and the skin and subjacent textures were dissected away from their attachments to the lower maxillary bone, until they were capable of being raised up to the desired position without the least stretching. This perpendicular incision was commenced at points nearly opposite the first molar tooth upon each side of the mouth, and extended from the margin of the orifice to a point fully half an inch beyond and around the base of the chin.

At this point of the operation, the mucous membrane, saved and dissected from the flaps removed at each angle of the mouth, was successfully utilized.

The bleeding having ceased and the chin substance being raised to its proper position as a lip, these thin flaps of mucous membrane were carefully turned around and secured to it with fine sutures of silk, forming in their joint extent a very respectable amount of inner lining. Elliptical incisions had been carried into the mucous membrane of each cheek, in order to allow these flaps being easily and properly turned into position.

The parts were now brought loosely together, and the patient placed in bed.

In the morning he was quite comfortable; the loose dressings were undone, and the raw and cut surfaces, from which all bleeding had ceased, were exposed. The teeth remaining in the back and uninjured portion of the lower jaw bone, three on one side and two on the other, stood elevated fully one and a half inches above the line of cut surface that covered what was remaining of the front and injured portion of the same bone. A large dental impression cup, especially contrived for the purpose, was filled with prepared plaster of Paris, and an accurate impression of the parts was obtained. From this impression, Dr. A. H. Fleming, dentist, by the evening of the same day, succeeded in making, in rubber, a very good substitute for the portion of jaw bone that had been shot away and which was surmounted by the proper number of artificial teeth. After the insertion of this plate, the parts were all brought securely together, the angle of the right side of the mouth being alone left open to permit of any future manipulation that might be necessary. One of the attendants had made, under my direction, a tight-fitting skull cap, and a stirrup, fitting accurately under the chin, furnished with a number of strong straps and buckles. After cropping the hair closely, these were applied, and by their means, the parts were kept in position. The healing took place rapidly and satisfactorily.—*Med. and Surg. Reporter.*

---

*Borate of Soda Gargles.*—A pharmacist of Lyons remarks that in gargles, borate of soda is generally combined with honey, or honey of roses, or even with syrup of mulberries ; that is to say, with products which dissolve a very small quantity of this salt. It would, therefore, be more advantageous to compound these measures with equal parts of glycerine and borate of soda. A definite and considerably more active solution is thus obtained ; for glycerine mixes well with honey, as well as with syrups.—*Med. and Surg. Reporter.*

---

*Quinine Gargle in Sore Throat.*—Dr. George Johnson gives the formula below as of excellent effect in sore throat, even severe diphtheritic cases :—

| | | |
|---|---|---|
| ℞ | Quiniæ sulphatis, | gr.xviij |
| | Acidi sulphurici diluti, | mxlij |
| | Aquæ, ad., | f ℥ vj. M |

He adds, I have found the quinine solution useful as a wash in aphthæ, stomatitis, and other affections of the mouth ; but my experience of it in these cases has been limited by the difficulty attending its use in childhood, owing to its very bitter taste.—*Med. and Surg. Reporter.*

*Andral for Glycosuria.*—In the *Gazette hebdomadaire* for April 16th, 1875, we find an analysis by Andral of eighty-four cases of glycosuria of which he had kept notes. It appears that the disease is very rare under 20 years of age, less rare between 20 and 30, most frequent between 40 and 50, little less so between 50 and 70, and very rare after the latter age. Of the eighty-four there were fifty-two males and thirty-two females. Various nervous affections seem to have an influence in developing or aggravating the glycosuria ; a deficiency of food amounting almost to starvation led to the disease in four cases. On the other hand, Andral had observed that many of these patients have been notably strong and vigorous before the development of the diabetes. An accidentally complicating disease will often cause a temporary diminution of the sugar eliminated ; as for ample an angina and a dysentery. A degree of hereditary influences was observed. While the general circulation ordinarily exhibits no lesions, the capillary circulation often does so, as, *e. g.*, in swelling and redness of the gums, injection of the conjunctiva, erythema of the skin and congestion of the lungs. There were four cases of gangrene among the eighty-four ; viz : once in the lungs, and three times in the feet and legs. The acid reaction of the saliva that has been observed may be due according to Andral, to an admixture with the ordinary mucus of the mouth. At the autopsies he constantly observed congestion of the liver and kidneys, attributable in the latter, at any rate, to excessive functional activity. There was also a peculiar induration and dryness of the spleen. Tubercular granulations were commonly fouud in the lungs, which Andral regards as secondary deposits due to the debility caused by the diabetes. No new points were brought out regarding treatment.—*Medical Record.*

---

*New Remedy for Burns.*—There has been in a hospital for many months a case of extensive burn, in which different applications have been tried. Every new dressing succeeded well f ora time, but soon it ceased to prove of advantage. The last agent that has been used, and is at present, is salicylic acid. The effect is more beneficial than that obtained by any of the former remedies. The method of using it is to form an emulsion with olive oil, one part of the salicylic acid to sixteen parts of oil. This mixture is painted over the ulcerated surface once or twice a day. It gives rise to a slight smarting sensation when first applied, but that soon passes off.—*The Pharmacist,* August, 1875.

---

*Makinq a New Nose out of a Finger.*—At the recent meeting of the British Medical Association, Dr. James Hardie, of Manchester, gave an account of a new rhinoplastic operation, which consisted in the substitution of the upper phalanx of the forefinger for the nasal bones and cartilages, so as to give the required nasal prominence. In the case of a young girl who had lost both nasal bones and cartilages, Dr. Hardie, after a failure of other methods, bandaged the arm in such a position as to enable the forefinger to be laid and plastered upon the nasal cavity, in which position the finger was kept for about three months. Gradually the finger became attached to the cavity, and ultimately the upper phalanx was separated from the rest by the forceps. Dr. Hardie's first intention was to employ the finger merely as a substitute for the nasal bones and cartilages, and to lay over it flaps of skin from the face or arm in the usual manner, and to this plan he ultimately returned, although at one time he was so satisfied with its appearance as to think of using the phalanx itself as a substitute for the nose.—*Boston Med. and Surg. Journal.*

---

*Applications to Burns.*—Mr. Charles Rice states that being requested to search for an application which would combine *transparency, cleanliness, body, rapidity of drying* and *flexibility*, he has succeeded in making the following combination, which has been used for more than a year in the hospitals of New York. "Take of the best *white glue* (extra) 15 ounces. Break it into small pieces, add to it 2 pints of cold water, and allow it to become soft. Then melt it on a water-bath, add to it fluid ounces of glycerine and 6 drachms of carbolic acid, and continue the heat on the water-bath until a *glossy, tough skin* begins to form over the surface in the intervals of stirring. The mixture may be used at once, after the glue is melted and the glycerin and carbolic acid are added, but when time allows, it is advisable to ged rid of a little more of the water, until the proper point is reached. On cooling, this mixture hardens to an elastic mass, covered with a shining parchment-like skin and may be kept for any time. When using it, it is placed for a few minutes on the water-bath until sufficiently liquid for application; (it should be quite fluid.) Should it at any time require too high a heat to become fluid, this may be corrected by adding a little water. It is applied by means of a broad brush, and forms in about two minutes a shining, smooth, flexible, and nearly transparent skin. It may be kept for any time, without spoiling, in delf or earthen dishes, or pots turned upside down. —*Medical News and Library.*

---

*Mode of Administering Ether.*—In the recent work of Dr. Robert B. Carter on the Eye, the writer refers to a plan adopted by Dr. Hood, of preceding the use of ether by a little chloroform, administering enough of the latter to impair the acuteness of sensation, but not enough to approach the confines of danger, and then completeing the anæsthesia by a full dose of ether given in the ordinary way, The method is not entirely new, and there are some cases, especially in children, in which it has decided advantages. One difficulty, however, is to determine where the confines of danger are located in the inhalation of chloroform.—*Pacific Medical and Surgical Journal.*

---

*Chloral as a Local Application for Ulcers.*—On visiting the wards of Guy's Hospital on the 7th of October, we saw several patients on whom a solution of hydrate of chloral had been used as a local application to ulcers, and the results appear to be sufficiently satisfactory to be worthy of record. Mr. Lucas commenced the use of chloral among his out-patients in August last, for cases of sloughing wounds and fetid ulcers, and being pleased with the result, he has since given it a somewhat extensive trial in the wards. The effect of the local application of chloral appears to be that of a powerful stimulant and disinfectant; it has no soothing or sedative effect upon the part to which it is applied, but, on the contrary, gives rise to considerable pain, which lasts some time ; nor does it, even when used over a very extensive surface, ever become absorbed in sufficient quantity to act as an hypnotic. Whether it is taken up into the circulation or not matters little, since the quantity used as local application is so small compared with the dose administered as an internal remedy, that, where the whole of the drug applied to find its way into the blood, the quantity absorbed would still be very much less than that of an ordinary sleeping draught. Its local application is, therefore, eminently safe and free from the dangers which sometimes follow the use of opium lotion or carbolic lotions long continued. Mr. Lucas has used solutions of various strengths, that which he has found most useful being a solution of four grains of hydrate of chloral in an ounce of water. The application of a lotion of this strength is, as we have just stated, often attended with considerable smarting which may last a quarter of an hour, but the smarting becomes less at each subsequent application. In cases where the patients have complained much of the smarting, the lotion has been diluted to the proportion of three or two grains to the ounce. The treatment of foul sloughing ulcers by means of chloral lotion has been attended with great success, the surface of the sore quickly cleansing and assuming a healthy appearance, whilst the subsequent healing has advanced with rapidity in some cases quite astonishing.—*Med. News and Library.*

*Preservation of Leeches.*—A concentrated solution of salicylic acid speedily destroys leeches, but a very weak solution preserves them in all their vigor. Four drops of a solution of a gramme of salicylic in 300 grammes of water to each 100 grammes of water is the strength recommended.—*Jour. de Chemie et de Pharmacie.*

---

*The Antiseptic Properties of Salicylic and Benzoic Acids.*—Prof. E. Salkowski, of Berlin, has been making some experiments on the relative antiseptic properties of salicylic and benzoic acids, adding these substances to albuminous solutions, and observing their condition subsequently as to turbidity or clearness, reaction, odor of putrefaction, and the occurrence of mould and bacteria. He concludes that salicylic acid in concentrated aqueous solutions can retard putrefaction, but cannot prevent it. He denies that it possesses deodorizing properties, as has been claimed, arguing that a deodorant action is to be explained in one of the following ways: either by the destruction of volatile emanations from the fluid, as permanganate of potash, chloride of lime, or sulphurous acid act; or by absorption, as in the case of charcoal, gypsum and the like, or by disguising the odor of decomposition, as does carbolic acid. He states that salicylic acts in no one of these ways, nor does he consider the assumption of Kolbe tenable, that it acts by breaking up into carbolic and carbonic acids. On the other hand, he declares that benzoic acid possesses much stronger antiseptic properties. He conjectures that the mode of preparation of this acid influences its antiseptic qualities, and recommends only that prepared from wine by the best makers. An other advantage is, that it is very much cheaper than salicylic acid. Our author considers both of these acids alike unfitted for internal use as antiseptics or antizymotics, because they are converted in the blood into soda salts. Neutral substances, or such as pass through the system unchanged, are to be preferred for this purpose. Among these, phenol, its substitution products, such as thymol, and the ethers of plienol, are pre-eminent.—*Med. Record.*

---

*Glass Cement.*—A cement to stop cracks in glass vessels, to resist moisture and heat, is made by dissolving caseine in a cold saturated solution of borax. With this solution, paste strips of hog or bullock's bladder, softened in water, on the cracks of glass, and dry at a gentle heat. If the vessel is to be heated, coat the bladder on the outside, just before it has become quite dry, with a paste of a rather concentrated solution of soda and quicklime or plaster of Paris.—*Exchange.*

